# Bond Graph Model of Cerebral Circulation: Toward Clinically Feasible Systemic Blood Flow Simulations

**DOI:** 10.3389/fphys.2018.00148

**Published:** 2018-03-02

**Authors:** Soroush Safaei, Pablo J. Blanco, Lucas O. Müller, Leif R. Hellevik, Peter J. Hunter

**Affiliations:** ^1^Auckland Bioengineering Institute, University of Auckland, Auckland, New Zealand; ^2^National Laboratory for Scientific Computing, Petrópolis, Brazil; ^3^National Institute of Science and Technology in Medicine Assisted by Scientific Computing, Petrópolis, Brazil; ^4^Division of Biomechanics, Department of Structural Engineering, Norwegian University of Science and Technology, Trondheim, Norway

**Keywords:** cardiovascular system, circulation model, bond graph, CellML, OpenCOR, ADAN model, 0D model, blood flow

## Abstract

We propose a detailed CellML model of the human cerebral circulation that runs faster than real time on a desktop computer and is designed for use in clinical settings when the speed of response is important. A lumped parameter mathematical model, which is based on a one-dimensional formulation of the flow of an incompressible fluid in distensible vessels, is constructed using a bond graph formulation to ensure mass conservation and energy conservation. The model includes arterial vessels with geometric and anatomical data based on the ADAN circulation model. The peripheral beds are represented by lumped parameter compartments. We compare the hemodynamics predicted by the bond graph formulation of the cerebral circulation with that given by a classical one-dimensional Navier-Stokes model working on top of the whole-body ADAN model. Outputs from the bond graph model, including the pressure and flow signatures and blood volumes, are compared with physiological data.

## 1. Introduction

Two challenges for biophysically based physiological modeling are to link the model parameters to patient-specific data and to make the models fast enough to become useful and accessible both, to reach a wide community of users, and to fit a clinical setting. For the prediction of pressure and flow in the patient-specific vascular system there is also the need to “close the loop” to ensure continuity of blood flow, and this requires a systems level model that includes arteries, veins and the capillary networks within specified tissue beds. The appropriate level of granularity for a model depends of course on the clinical or scientific question being studied. Available formulations for blood flow include three-dimensional (3D) FSI models (Heil and Hazel, 2011; Brown et al., 2012), rigid domain 3D fluid models (Shojima et al., 2004; Cebral et al., 2005), one-dimensional (1D) models (Reymond et al., 2009; Blanco et al., 2014), and zero-dimensional (0D) or “lumped-parameter” models (Korakianitis and Shi, 2006). In this paper we address the issue of execution time and the question of granularity in the context of a model of the cerebral circulation which will make it possible to model the exchange of solutes between blood and various tissue beds under conditions where vasodilation can also occur.

The analysis of pressure and flow in the vascular system is usually based on the incompressible direct Navier-Stokes (DNS) equations that ensure mass conservation and energy balance. We assume laminar flow since the Reynolds numbers are below the transition to turbulence in our example. The model parameters for the incompressible fluid considered here, blood, viscosity, and density, are both well understood and measurable. The compliance of the vessel wall is described by a constitutive relation that links the vessel diameter (and temporal deformation rate in the case of viscoelastic vessel wall models) to the fluid pressure. 1D blood flow equations are derived from 3D DNS by assuming negligible radial flow and integrating axial fluid velocity over the vessel cross-section. Moreover, an additional assumption must be made about the time-varying radial profile of the axial velocity. For a steady state well developed flow, such profile is of course parabolic, but a more realistic assumption is a flatter-than-parabolic profile, which then requires at least one more empirical parameter to be specified (Hunter, 1972). These empirically determined parameters imply that, given the uncertainty in geometrical and biophysical parameters for the patient specific modeling, there is always uncertainty in the predicted pressure and flow results and the need to include computationally expensive fluid calculations (e.g., solving 3D DNS) must be balanced against this uncertainty.

The primary goal of this paper is to compare flow and pressure waveforms predicted by a 1D blood flow model, consisting of partial differential equations, with the output of a bond graph based model, which generates a system of ordinary differential equations (ODEs) that can be solved approximately 200x faster than the 1D model and at close to real time on a desktop computer. The model used here is based on the ADAN cerebral circulation model (Blanco et al., 2015), along with a relatively simple model of flow through the heart and lungs, as an example. The results show the bond graph solution to be within 5% of the 1D model solution for flow and pressure at every point in the cerebral circulation model.

This paper is organized as follows. In section 2.1, the bond graph method is introduced and various components are presented. In section 2.2, the architecture of the cardiovascular system model is described. The software, model structure and simulation setup are presented in the section 3.1. The simulation results of the bond graph arterial model (open-loop) and comparisons against the 1D model are presented in section 3.2 and section 3.3. Then the simulation results for the closed-loop bond graph model of the cardiovascular system are demonstrated in section 3.4. Finally, concluding remarks and future works are outlined in section 4.

## 2. Materials and methods

### 2.1. Bond graph approach

The bond graph approach to formulating models dealing with mass and energy transfer was developed by Henry Paynter in the 1960s to represent electro-mechanical control systems (Paynter, 1961). It was later extended to include chemical processes by Breedveld (1984), including concepts from the theory of network thermodynamics by Aharon Katchalsky and colleagues (Oster et al., 1971). Papers by Peter Gawthrop and Edmund Crampin have brought the approach into the bioengineering domain (Gawthrop and Crampin, 2014; Gawthrop et al., 2015a,b; Gawthrop and Crampin, 2016).

The first key idea, based on recognizing that energy and power are the only quantities that are common across different physical systems, is to separate energy transmission from storage and dissipation, and to provide the concept of *potential* (called “effort” in the engineering literature) with units of Joules per some_quantity as the common driving force behind the *flow* of that some_quantity per second. The product of potential and flow is then always power in units of Joules per second. The “some_quantity” has units of meters, meters^3^, Coulombs, Candela, moles, or entropy for, respectively, rigid body mechanics, continuum mechanics (including fluid flow), electrical, electromagnetic, chemical, and heat transfer processes. As explained further below, the second key concept is that of a *0-junction*, where potential is defined and mass balance is applied, and a *1-junction*, where flow is defined and energy balance is applied. The extraordinary utility of these concepts is to recognize that Kirchhoff's voltage law in electrical circuits, Newton's force balance in a mechanical system, and stoichiometric balance in a biochemical system, are all just different manifestations of the same underlying principle of energy conservation and can therefore be represented by the same bond graph equation.

#### 2.1.1. Units

Many physical systems can be described by a driving *potential* expressed as Joules per unit of some quantity, and a *flow* expressed as that quantity per second. The quantity could be coulomb, meters, moles, etc., in different physical systems. The power is always the product of the driving force and the flow expressed as Joules per second. The seven units of the SI system under the newly proposed definitions are now based on constants that are consistent with the use of Joules and seconds (together covering energy and power), meters, moles, entropy, Coulombs, and Candela. Table 1 in Supplementary Material displays the bond graph concepts in the fluid mechanics domain.

#### 2.1.2. Bond graph formulation

**Figure 1:**
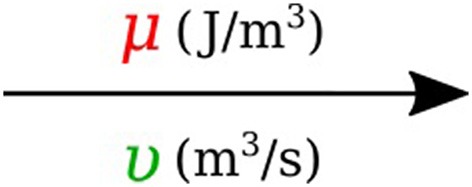
Representation of energy bond.

In bond graph formulation, there are four basic variables. In the fluid mechanics domain these are given by: potential μ is energy density or *pressure* (J.m^−3^), flow υ is *volumetric flow* (m^3^.s^−1^), time integral of potential *p* is *momentum* (J.s.m^−3^) and time integral of flow *q* is quantity or *volume* (m^3^). Product μ.υ is power (J.s^−1^) which is a generalized coordinate to model the complete systems residing in several energy domains. A bond with covariables μ and υ is therefore used to represent *transmission of energy*. The bond represents a mechanism for the transmission of energy and power, and the arrow head indicates the assumed direction of power flow (see Figure 1). The flow υ and potential μ must satisfy conservation laws.

There are also the concept of *0-junction* and *1-junction* for conservation laws. The *0-junction* defines a common potential μ which ensures that the potential is identical at each port and imposes mass conservation constraint based on υ. The *1-junction* defines a common flow υ which ensures that the flow is identical at each port and imposes energy conservation constraint based on μ. Since sum of the flows is zero with μ constant for *0-junction* and sum of the potentials is zero with υ constant for *1-junction*, the transmission of power through junction is conserved for both kinds of junctions, that is:

(1) ∑μ.υ=0.

#### 2.1.3. Bond graph elements

Bond graph formulation is a graphical notation for the set of linear constraint equations (the conservation laws), but the constitutive relations (to be addressed next) can be nonlinear.

##### 2.1.3.1. *R*-element

Energy μ can be dissipated by a resistor *R* in proportion to the flow υ with an empirical relation which can be a simple linear relation such as Equation 2 or a complex nonlinear relation:

(2) μ=υR.

In the fluid mechanics systems, the *R*-element represents the viscous resistance in opposition to the blood flow and for a cylindrical vessel can be analytically calculated using Poiseuille relation:

(3) $$R=\frac{8\nu l}{\pi r^{4}},$$

where ν is the blood viscosity, *l* is the vessel length and *r* is the vessel radius.

##### 2.1.3.2. *C*-element

Energy μ can be stored statically by a capacitor *C* without any loss. In the bond graph terminology, a one-port capacitor relates energy to the quantity *q* or time integral of flow by an empirical relation such as Equation 4. The *C*-element stores *q* by accumulating the net flow υ to the storage element:

(4) $$\mu =\frac{q}{C},$$

(5) $$\dot{q}=\upsilon ,$$

in which the dot stands for time derivative. In the fluid mechanics systems, and particularly in the cardiovascular system, the *C*-element represents the vessel wall compliance and can be calculated from blood vessel properties. For a homogeneous linear elastic material and for a cylindrical vessel, the compliance is characterized as follows:

(6) $$C=\frac{2\pi r^{3}l}{hE},$$

where *E* is the Young's modulus and *h* is the vessel thickness.

##### 2.1.3.3. *I*-element

Energy μ can be stored dynamically by an inductor *I* without any loss. In bond graph formulation, a one-port inductor relates flow to the momentum *p* or time integral of potential by an empirical relation such as Equation 7. The *I* stores *p* by accumulating the net potential μ to the storage element.

(7) $$\upsilon =\frac{p}{I},$$

(8) $$\dot{p}=\mu .$$

In the fluid mechanics systems, the *I*-element is used to model the mass inertial effects in a pipe and can be defined for straight cylindrical vessels as:

(9) $$I=\frac{\rho l}{\pi r^{2}},$$

where ρ is the blood density and *l* is the vessel length.

**Figure 2:**
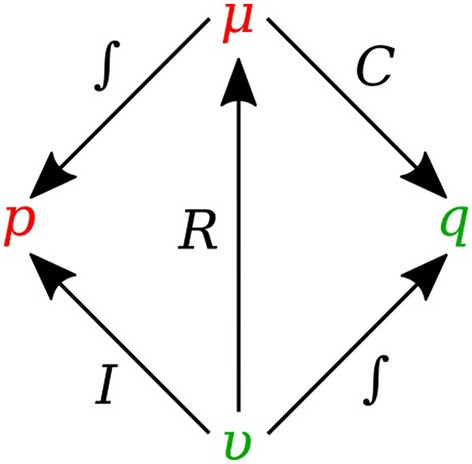
State variables and constitutive relations in the bond graph approach.

Figure 2 shows the relation of the state variables to the constitutive relations.

#### 2.1.4. Causality

**Figure 3:**
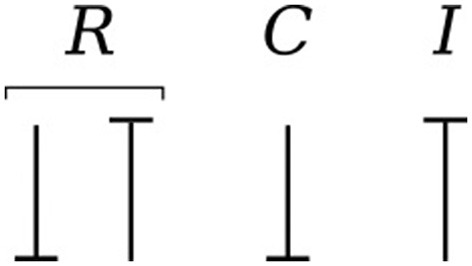
Preferred causality for *R*, *C*, and *I* elements.

Causality establishes the cause and the effect relationship. It specifically implies that either the potential or flow variable on that bond is known. Causality is generally indicated by a causal stroke at the end to which the potential receiver is connected. Elements which store or dissipate energy do not impose causality on the system, but they have preferred causality for computational reasons. These elements with their preferred causality are shown in Figure 3.

**Figure 4:**
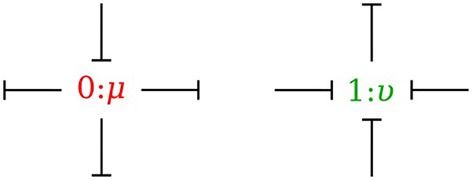
Causality in four-port 0-junction and 1-junction.

In the bond graph approach, junctions interconnect the corresponding elements and constrain the possible causalities of the element ports connected to it. A *0-junction* can only have one potential output. In a similar way, a *1-junction* can only have one flow output. Figure 4 illustrates causality in four-port *0-junction* and *1-junction*.

#### 2.1.5. Vessel segments

**Figure 5:**
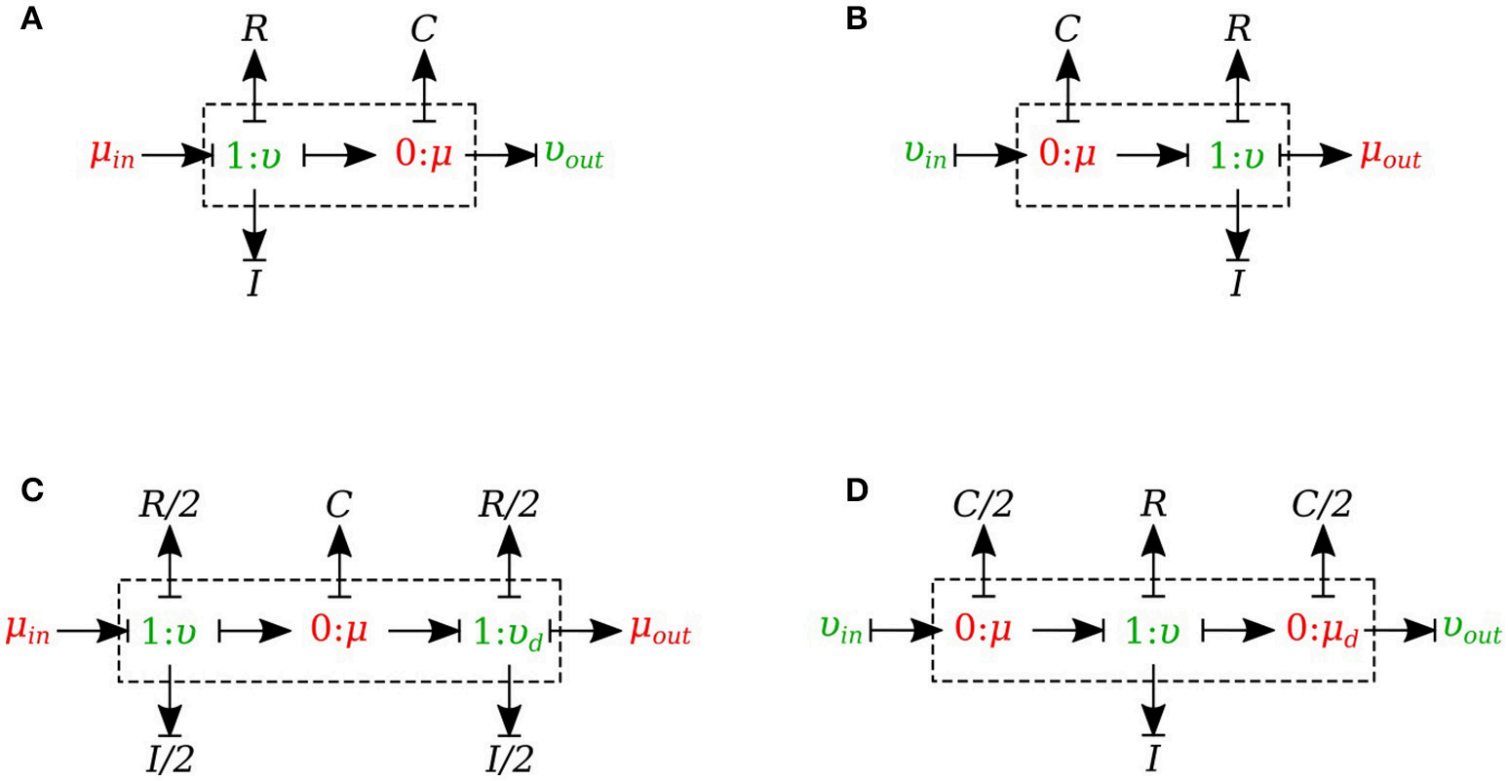
Different configurations of bond graph model for a vessel segment, **(A)** the μυ-type has inlet pressure BC and outlet flow BC, **(B)** the υμ-type has the reversed characteristics, **(C)** coupling these two configurations with the *C* in the middle gives us a μμ-type with inlet and outlet pressure BCs. The *R* and *I* values are divided equally between the two resistors and two inductors at both ends, **(D)** coupling these two configurations with the *R* and *I* in the middle gives us a υυ-type with inlet and outlet flow BCs. The *C*-value is divided equally between the two capacitors at both ends.

In this section, we developed a library of bond graph elements for modeling the blood flow in distensible vessels. The modularity of the bond graph approach enables us to develop a wide range of elements and incorporate them into the model based on a set of assumptions. There are four basic types of elements for a vessel segment depending on whether potential or flow BC is prescribed at the inlet and the outlet (see Figure 5). Each vessel segment is represented by a parallel combination of one *C*-element and a series combination of one *R*-element and one *I*-element. The *C*, *R*, and *I* represent the vessel wall compliance, the viscous friction and the inertia of the blood, respectively. These elements are interconnected by a *0-junction* for same blood pressure and a *1-junction* for the same blood flow.

The set of equations for *μυ-type* (see Figure 5A) after rearrangements is:

(10) $$\dot{\mu }=\frac{\upsilon -\upsilon_{out}}{C},$$

(11) $$\dot{\upsilon }=\frac{\mu_{in}-\mu -\upsilon R}{I}.$$

Also a similar set of equations can be derived for *υμ-type* (see Figure 5B). For *μμ-type* (see Figure 5C) we have:

(12) $$\dot{\mu }=\frac{\upsilon -\upsilon_{d}}{C},$$

(13) $$\dot{\upsilon }=\frac{\mu_{in}-\mu -\upsilon \left(R/2\right)}{I/2},$$

(14) $${\dot{\upsilon }}_{d}=\frac{\mu -\mu_{out}-\upsilon_{d}\left(R/2\right)}{I/2}.$$

In a similar way, we can write the equations for *υυ-type* (see Figure 5D).

#### 2.1.6. Viscoelastic vessel wall

**Figure 6:**
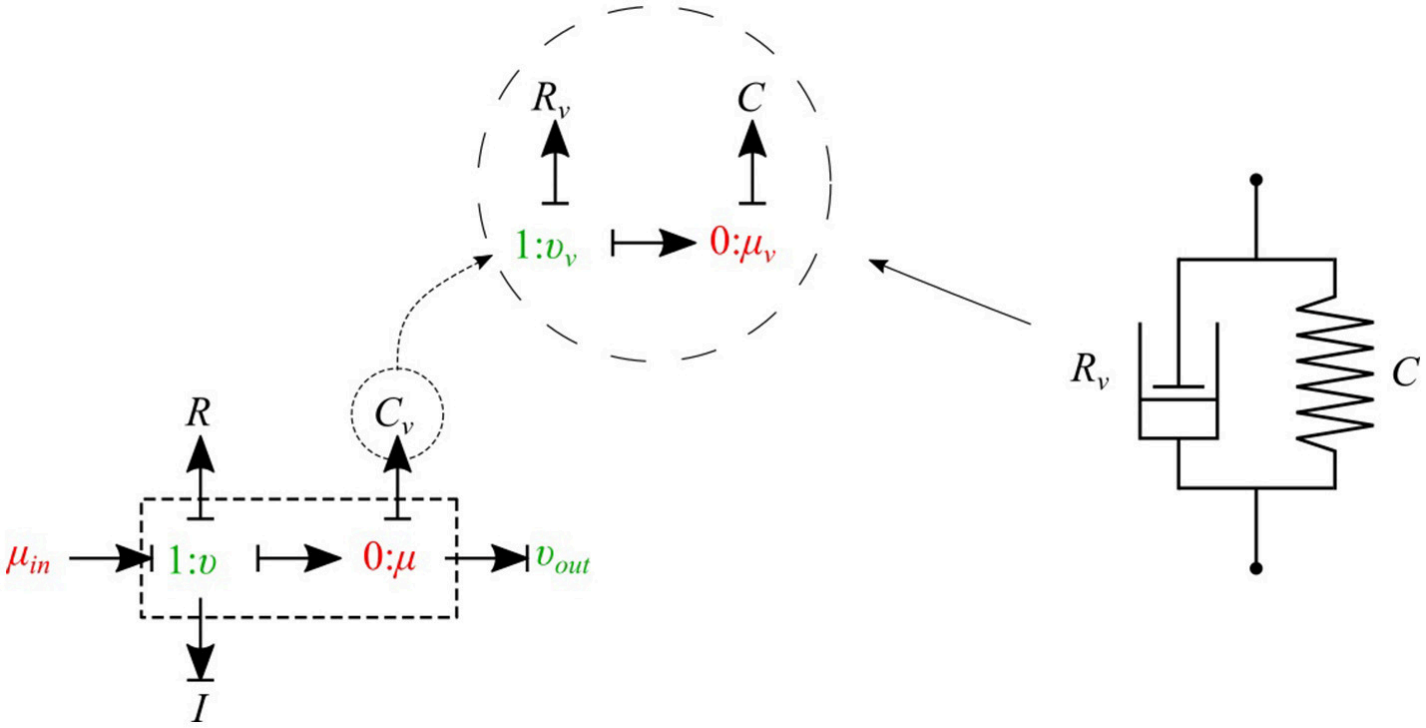
Mechanical representation of the Voigt model consisting of a parallel arrangement of a spring *C* and a dash pot *R*_*v*_, which represent elastic, and viscous material behavior, respectively.

The bond graph representation makes it very easy to implement the viscoelasticity effect of the vessel wall into the model. Two common existing models in the literature are the Voigt model and the Maxwell model. A more sophisticated model is the generalized model developed by Westerhof and Noordergraaf (1970). However, the generalized model is complex and computationally expensive to solve, and for this reason we chose the Voigt model to represent the viscoelastic effect of the vessel wall in this work. The classical Voigt model in mechanical symbols is shown in Figure 6.

Bond graph representation of the Voigt model is illustrated in Figure 6. By taking advantage of the modular nature of the bond graph technique we can easily plug in the Voigt model into any configuration of the vessel elements described before. Figure 6 shows the viscoelastic *μυ-type* element. The governing equations for this element are described below:

(15) $$\mu =\mu_{v}+\left(\upsilon -\upsilon_{out}\right)R_{v},$$

(16) $${\dot{\mu }}_{v}=\frac{\upsilon -\upsilon_{out}}{C},$$

(17) $$\dot{\upsilon }=\frac{\mu_{in}-\mu -\upsilon R}{I}.$$

As can be seen, by adding only one equation to the basic set of equations we can take into account the viscoelastic effect of the vessel wall. Using a similar approach, other basic elements can also be equipped with the viscoelastic effect accounted for by *C*_*v*_.

#### 2.1.7. Junctions

The *0-junction* is a powerful concept in the bond graph approach that allows us to model the splitting or merging flows in blood vessels. It satisfies the conservation of flow and also imposes a common potential on all the branches to make sure pressure is continuous throughout the junction, which is a good approximation of branching in arterial vessels. It is important to know that only *μυ-type* and *υυ-type* elements can be used as the parent vessel in a junction. In a similar way, only *μυ-type* and *μμ-type* elements can be implemented as daughter vessels in a junction. These restrictions are due to arranging compatible segment types into a structure, with inlets and outlets coupled appropriately in the sense that BCs are settled by the state of their adjacent compartments.

##### 2.1.7.1. Splitting flow

**Figure 7:**
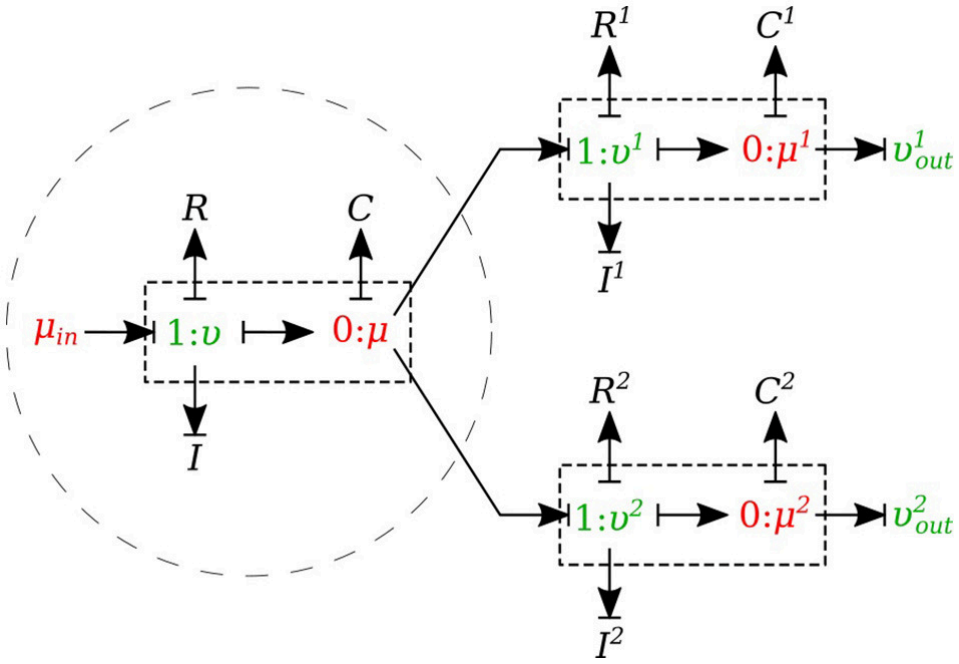
Bond graph model for a bifurcating branch.

In the splitting flow junctions, a *0-junction* represents the separation point at the end of the parent vessel and the daughter vessels are connected via this port to the parent vessel (see Figure 7).

We created another element specifically for splitting flow junctions and called it *μυ-split-type*. To implement the splitting flow junction, only the parent vessel element needs to use *μυ-split-type* and the daughter vessels remain basic *μυ-type*. The governing equations for this element type are stated below:

(18) $$\dot{\mu }=\frac{\upsilon -\upsilon^{1}-\upsilon^{2}}{C},$$

(19) $$\dot{\upsilon }=\frac{\mu_{in}-\mu -\upsilon R}{I},$$

where υ^1^ and υ^2^ are the daughter branches flow.

##### 2.1.7.2. Merging flow

**Figure 8:**
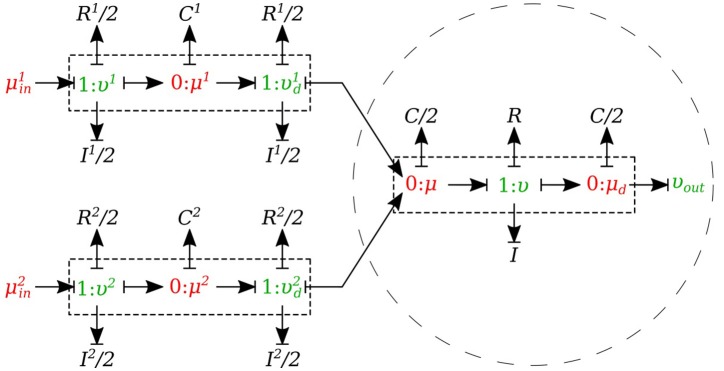
Bond graph model for a merging branch.

In the merging flow junctions, a *0-junction* represents the adjoining point at the beginning of the parent vessel and the daughter vessels are connected via this port to the parent vessel (see Figure 8).

We created another element specifically for merging flow junctions and called it *υυ-merge-type*. To implement the merging flow junction, only the parent vessel element needs to use *υυ-merge-type* and the daughter vessels remain basic *μμ-type*. The governing equations for this element type are stated below:

(20) $$\dot{\mu }=\frac{\upsilon_{d}^{1}+\upsilon_{d}^{2}-\upsilon }{C/2},$$

(21) $$\dot{\upsilon }=\frac{\mu -\mu_{d}-\upsilon R}{I},$$

(22) $${\dot{\mu }}_{d}=\frac{\upsilon -\upsilon_{out}}{C/2},$$

where $\upsilon_{d}^{1}$ and $\upsilon_{d}^{2}$ are the flows through the daughter branches.

#### 2.1.8. Peripheral circulation

**Figure 9:**
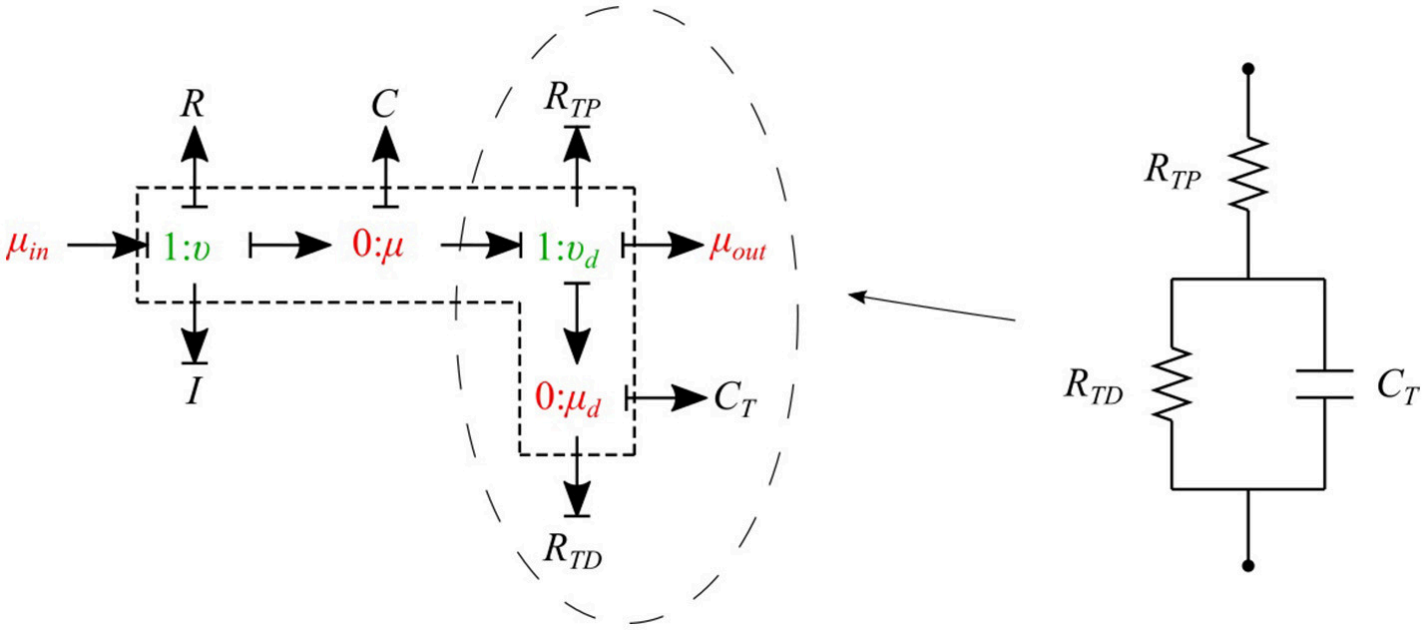
Bond graph model for an RCR terminal.

The cumulative effects of all distal vessels (small arteries, arterioles, and capillaries) at terminal locations of the truncated arteries are modeled using *RCR* Windkessel elements (Westerhof et al., 1969; Stergiopulos et al., 1992). For this purpose, a bond graph model of the *RCR* element is developed and attached to a *μυ-type* element to create a special bond graph element *μμ-BC-type* for terminal vessels. *RCR* element contains a proximal terminal resistance *R*_*TP*_ in series with a parallel arrangement of a terminal capacitor *C*_*T*_ and a distal terminal resistance, *R*_*TD*_ (see Figure 9).

The governing equations for the *μμ-BC-type* element are:

(23) $$\dot{\mu }=\frac{\upsilon -\upsilon_{d}}{C},$$

(24) $$\dot{\upsilon }=\frac{\mu_{in}-\mu -\upsilon R}{I},$$

(25) $${\dot{\mu }}_{d}=\frac{\upsilon_{d}-\frac{\mu_{d}}{R_{TD}}}{C_{T}},$$

(26) $$\upsilon_{d}=\frac{\mu -\mu_{d}-\mu_{out}}{R_{TP}}.$$

### 2.2. Cardiovascular system

The cardiovascular system is composed of three parts - heart, systemic circulation loop, and pulmonary circulation loop. In this section, we briefly explain how these components are modeled using the bond graph approach. Table 2 in Supplementary Material shows the bond graph elements that have been developed for modeling blood flow in the cardiovascular system. Based on the assumptions and locations, we import vessel segments with the appropriate element type as a new module and connect it to the system.

#### 2.2.1. Pulmonary circulation

**Figure 10:**
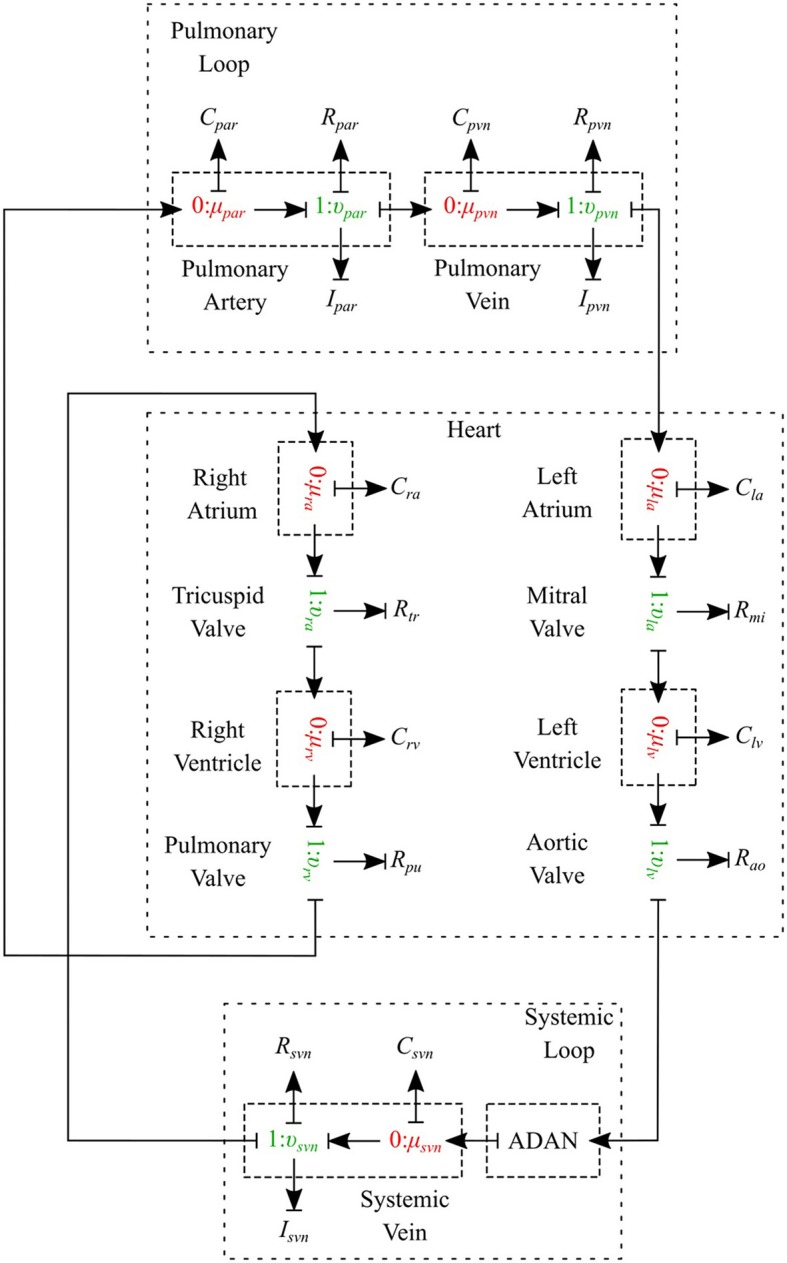
Schematic of the cardiovascular system.

The pulmonary circulation is modeled as described in Blanco and Feijóo (2013). We divide it into 2 main compartments, arteries (*par*) and veins (*pvn*). The arteries are highly elastic and the flow is pulsatile in these compartments, so the resistance, compliance and inductance effects must be considered and υμ-type is the most suitable bond graph element based on the prescribed BCs. Veins also are modeled using the υμ-type element (see Figure 10).

#### 2.2.2. Systemic circulation

**Figure 11:**
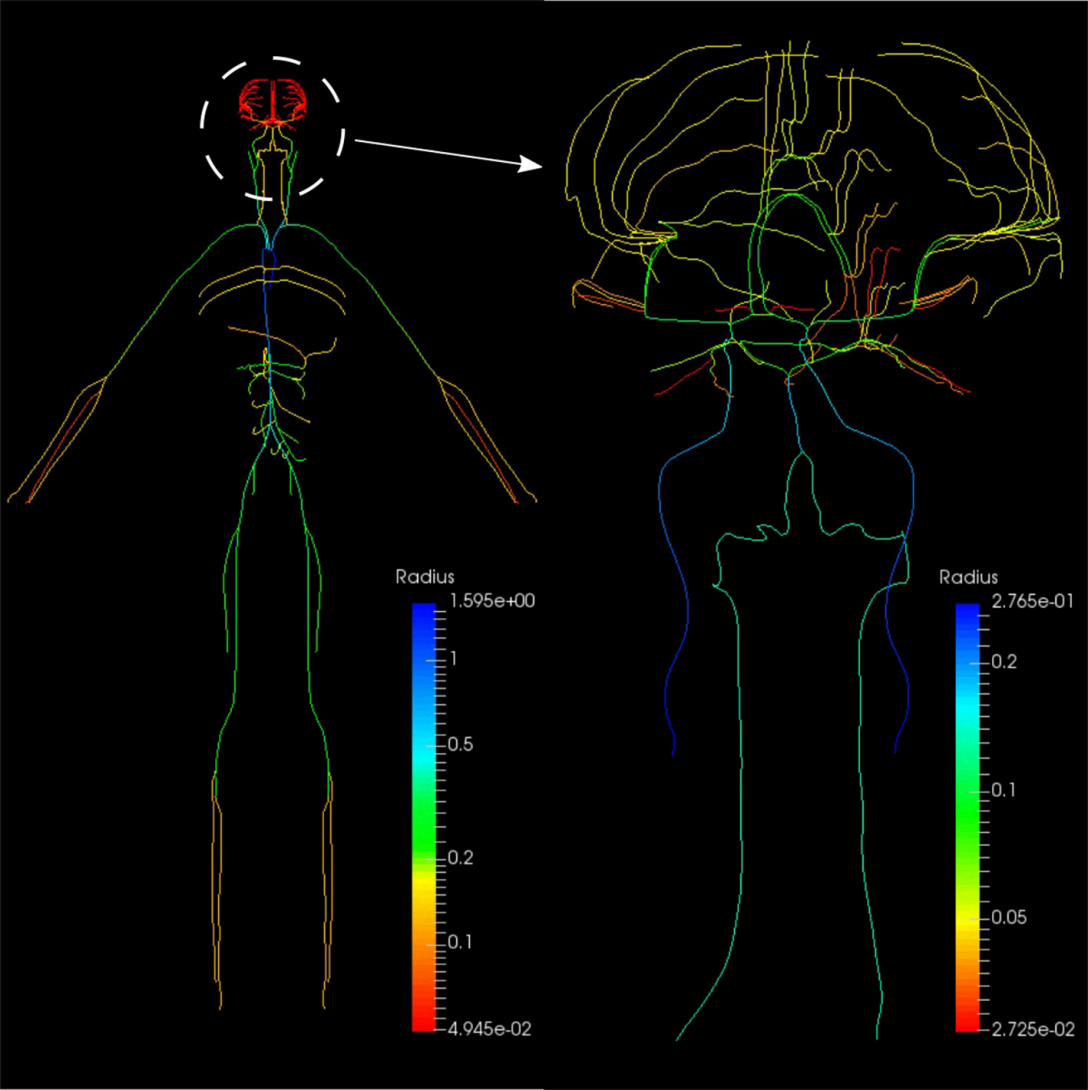
Anatomically Detailed Arterial Network (ADAN) model with detail of the cerebral vasculature. The varying colors represent the vessel radii.

The systemic circulation loop consists of a reduced version of the ADAN model Blanco et al. (2014, 2015) and the veins compartment which is similar to the pulmonary veins compartment. Such a reduced version of the ADAN model is composed of a 218-segment arterial model which consisted of the integration between the ADAN-86 model (Safaei et al., 2016) and the anatomically detailed cerebral vasculature of the ADAN model Blanco et al. (2015). Figure 11 displays the entire model with a detail of the cerebral vasculature.

**Figure 12:**
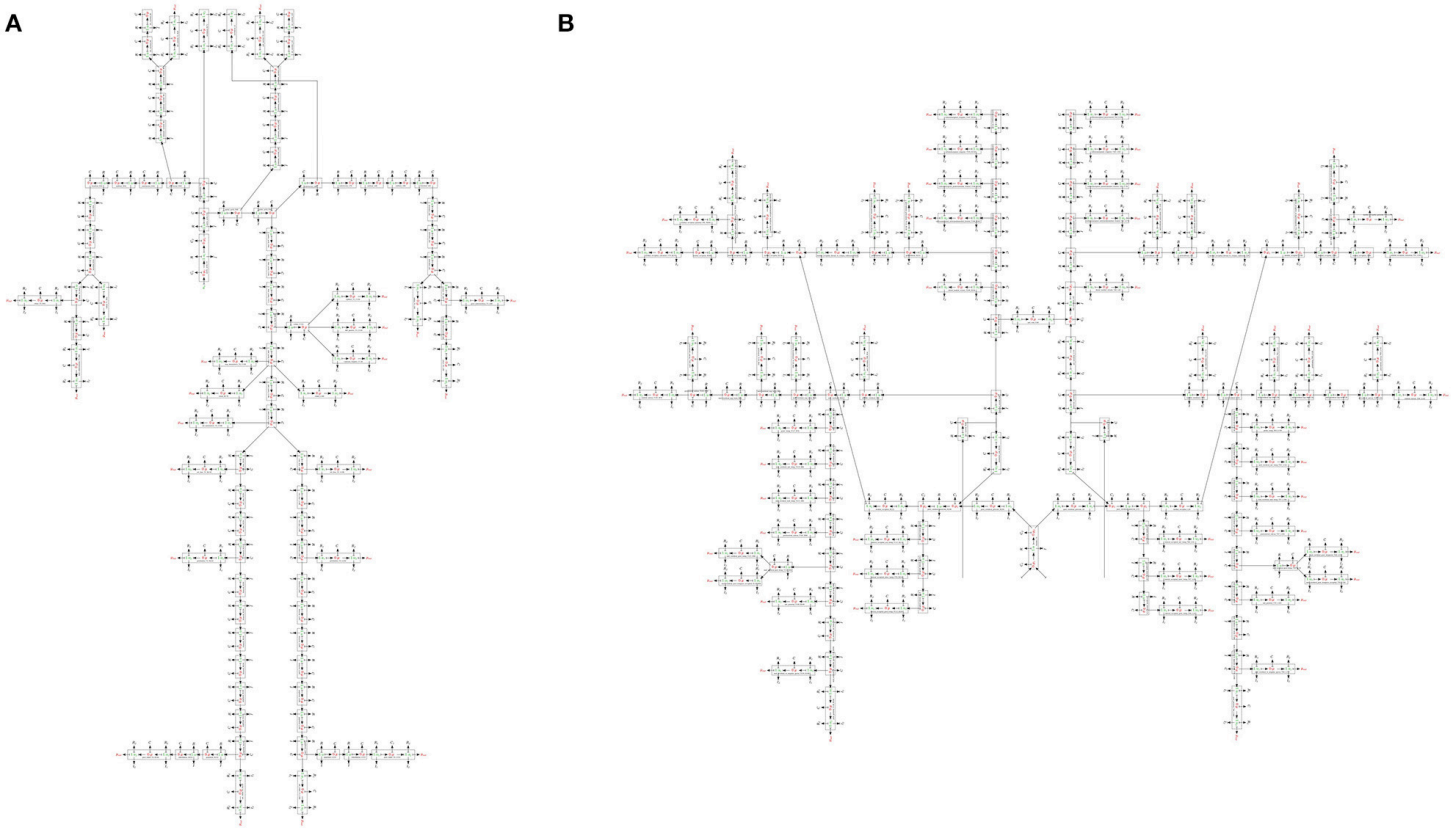
**(A)** The bond graph model of the ADAN-86 model, **(B)** the bond graph model of the ADAN-brain model. These two models are detached in this figure for clarity.

We constructed a bond graph model using the geometrical properties of the ADAN model (i.e., vessel radius and wall thickness). Figure 12A,B illustrate the bond graph model of the systemic arteries and cerebral circulation, respectively.

#### 2.2.3. Heart

The heart is modeled as a four-chamber pump with variable elastance and four valves: tricuspid valve, pulmonary valve, mitral valve and aortic valve. The basic pressure-flow relation in each valve is represented with an orifice model which is an advancement over the diode models (Korakianitis and Shi, 2006). The left and right atria and ventricles are modeled as capacitors with time-varying elastance which is a function of the characteristic elastance and an activation function. The activation function represents the contraction and relaxation changes in each cardiac chamber (see Figure 10).

##### 2.2.3.1. Ventricles

The left ventricle is represented by a special type of *C*-element which has a time-varying compliance function *C*_*lv*_(*t*). υ_*la*_ and υ_*lv*_ represent flow through the mitral and aortic valves, respectively, *q*_*lv*_ is the blood volume and μ_*lv*_ is the blood pressure inside the left ventricle. The differential equations governing the left ventricle model are as follows:

(27) $${\dot{q}}_{lv}=\upsilon_{la}-\upsilon_{lv},$$

(28) $$\mu_{lv}=\frac{q_{lv}-q_{lv}^{o}}{C_{lv}(t)},$$

where $q_{lv}^{o}$ refers to the dead volume of the chamber. The time-varying compliance function *C*_*lv*_(*t*) is the inverse of time-varying elastance function *E*_*lv*_(*t*) and it has been used so as to be consistent with the basic *C*-element constitutive relation:

(29) $$C_{lv}(t)=\frac{1}{E_{lv}(t)}.$$

*E*_*lv*_(*t*) is a function of the characteristic elastance and an activation function *e*_*v*_(*t*):

(30) $$E_{lv}(t)=E_{lv}^{B}+e_{v}(t)E_{lv}^{A}.$$

Here, $E_{lv}^{A}$ and $E_{lv}^{B}$ are the amplitude and baseline values of the elastance, and *e*_*v*_(*t*) is the ventricle activation function and expresses the contraction and the relaxation changes in the ventricular muscle:

(31) $$e_{v}(t)=\left\{ \begin{array}{l} \frac{1}{2}\left[1-\cos\left(\pi \frac{t}{T_{vc}}\right)\right],\;0\leq t\leq T_{vc}\\ \frac{1}{2}\left[1+\cos\left(\pi \frac{\left(t-T_{vc}\right)}{T_{vr}}\right)\right],\;T_{vc}<t\leq T_{vc}+T_{vr}\\ 0,\;T_{vc}+T_{vr}<t\leq T \end{array}\right.$$

where *T* is the duration of a cardiac cycle. *T*_*vc*_ and *T*_*vr*_ represent the durations of contraction and relaxation of the ventricles, respectively. The right ventricle is also modeled in a similar manner to the left ventricle model, with different values for system parameters (see Table 3 in Supplementary Material).

##### 2.2.3.2. Atria

The bond graph model of the atrium is also developed in a similar way to that of the ventricle. The only difference is that the atrium activation function which expresses the contraction and the relaxation changes in the atrial muscle. For the left atrium *e*_*a*_(*t*) is:

(32) $$e_{a}(t)=\left\{ \begin{array}{l} \frac{1}{2}\left[1+\cos\left(\pi \frac{\left(t+T-t_{ar}\right)}{T_{ar}}\right)\right],\;0\leq t\leq t_{ar}+T_{ar}-T\\ 0,\;t_{ar}+T_{ar}-T<t\leq t_{ac}\\ \frac{1}{2}\left[1-\cos\left(\pi \frac{\left(t-t_{ac}\right)}{T_{ac}}\right)\right],\;t_{ac}<t\leq t_{ac}+T_{ac}\\ \frac{1}{2}\left[1+\cos\left(\pi \frac{\left(t-t_{ar}\right)}{T_{ar}}\right)\right],\;t_{ac}+T_{ac}<t\leq T \end{array}\right.$$

where *T*_*ac*_ and *T*_*ar*_ are the durations of contraction and relaxation of the atria, and *t*_*ac*_ and *t*_*ar*_ represent the times when the atria start to contract and relax, respectively. An analogous relation applies to the right atrium activation function.

##### 2.2.3.3. Valves

Heart valves are modeled by a special type of *R*-element which instead of the conventional constitutive relation (Equation 2), uses a nonlinear pressure-flow relation of the orifice model. For the aortic valve we have:

(33) $$\upsilon_{lv}=R_{ao}\alpha_{ao}\sqrt{\lfloor \mu_{lv}-\mu_{root}\rfloor },$$

where υ_*lv*_ is the blood flow through the aortic valve, μ_*lv*_ is the blood pressure inside the left ventricle, μ_*root*_ is the blood pressure in the aortic root, *R*_*ao*_ is the aortic valve resistance, and α_*ao*_ is the aortic valve opening coefficient. Depending on which side of the valve has higher pressure, the coefficient α_*ao*_ can switch between fully closed and fully open states:

(34) $$\alpha_{ao}=\{\begin{array}{ll} 1, & \mu_{lv}>\mu_{root}\\ 0, & \mu_{lv}\leq \mu_{root} \end{array}$$

The rest of the valves are modeled in the same way with different system parameters (see Table 3 in Supplementary Material).

#### 2.2.4. Physiological data

The geometrical parameters of the 218 arteries were prescribed based on the data reported in Blanco et al. (2015). Vessel wall thickness *h* is calculated using the following relation:

(35) $$h=r_{o}(ae^{br_{o}}+ce^{dr_{o}}),$$

where *r*_*o*_ is the lumen radius. *a*, *b*, *c*, and *d* are the fitting parameters (see Table 3 in Supplementary Material). The elastic modulus of the arteries were calculated from Blanco et al. (2015). The viscoelastic wall properties are calculated using the relationship between the Voigt model components (Westerhof and Noordergraaf, 1970):

(36) $$R_{v}=\frac{f}{C},$$

where *R*_*v*_ is the viscous damping of the wall, *C* is the vessel wall compliance evaluated using Equation 6, and *f* is the time constant for stress relaxation (see Table 3 in Supplementary Material). The peripheral resistances (*R*_*TP*_ and *R*_*TD*_) and compliances (*C*_*T*_) were derived from Blanco et al. (2015). The parameters used in the heart, pulmonary loop and venous system have been assigned or estimated based on the data reported in Liang et al. (2009) and Blanco and Feijóo (2013). The cardiac valve model and the parameters have been adopted from Korakianitis and Shi (2006) (see Table 3 in Supplementary Material).

## 3. Simulations and results

### 3.1. OpenCOR simulation

OpenCOR (opencor.ws) is an open source modeling environment that works on Windows, Linux and OS X and can be used to organize, edit, simulate and analyse models of ODEs or differential algebraic equations encoded in the CellML format (Garny and Hunter, 2015). It relies on a modular approach, which means that all of its features come in the form of plugins. The bond graph model of the cardiovascular system has been developed using OpenCOR in four separate CellML files:

#### 3.1.1. BG_Modules.cellml

This file is the bond graph library and the elements listed in Table 2 in Supplementary Material including all the governing equations exist in this file. The modules defined in this file can be imported into the main file to represent a specific vessel segment or any other element in the fluid mechanics domain.

#### 3.1.2. Parameters.cellml

All the parameters have been defined in this file. These parameters include the geometric properties of all the ADAN vessels (length, radius, thickness, Young's modulus), resistance and capacitance at the ADAN terminal locations, and all the system parameters for the heart and the pulmonary circulation path models.

#### 3.1.3. Units.cellml

The basic units are implemented in CellML inherently, while any alternative unit system required needs to be constructed. This file contains all the constructed units in the bond graph approach that have been used in the present model.

#### 3.1.4. Main.cellml

This is the executable file which runs the simulations using OpenCOR software. It imports all the required modules and contains the information about the model structure, elements connectivity, and mappings between different components.

**Figure 13:**
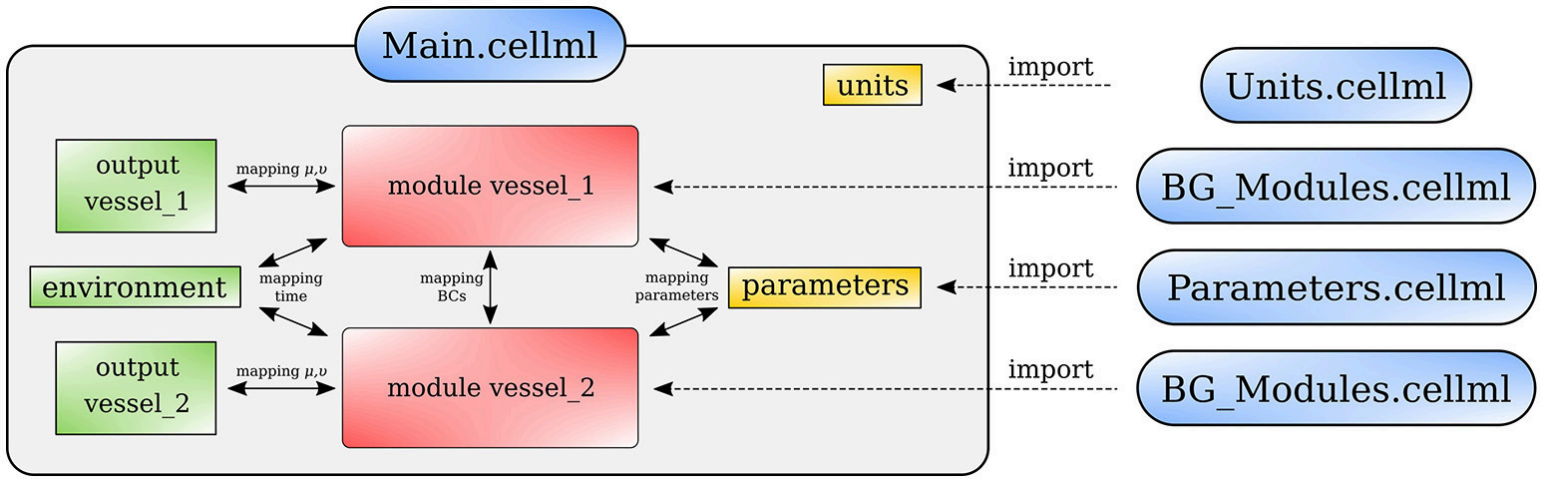
Overall structure of the cardiovascular system model showing the CellML model imports and the other key parts (units, components, and mappings) of the top level CellML model.

Figure 13 shows schematically how components are connected and communicate with each other. The full model is made publicly available at https://models.physiomeproject.org/workspace/4ac.

### 3.2. One-dimensional ADAN model

As stated at the beginning of this work, one of the primary goals in this contribution is to provide a comparison of the predictions delivered by the bond graph model and the predictions of the complete ADAN model. The latter is therefore regarded as a reference solution in the present context, and will be referred simply as the “ADAN solution.”

The ADAN model incorporates 2, 142 arterial vessels (yielding overall over 4, 000 arterial segments), 1, 598 of which have a well determined name according to the Anatomical Terminology. The disposition of these vessels correspond to a generic male individual of approximately 1.7 m in height. The model supplies blood to 28 specific organs, plus the supply to 116 vascular territories which accommodate the distributed organs. Each of these territories packs the bones, nerves, muscles, fascia and skin. The ADAN model also includes the additional vessels reported in Blanco et al. (2016). As for the calibration, peripheral resistances are determined according to the peripheral blood flow distribution reported in Blanco et al. (2014), while the calibration of arterial vessel behavior (including elastin and collagen constituents as well as viscoelastic phenomena) follow Blanco et al. (2015). The inflow condition is defined below. Finally, blood density and viscosity are taken to be μ = 0.004 J.s.m^−3^ and ρ = 1040 J.s^2^.m^−5^. Finally, the 1D equations for modeling the flow of an incompressible fluid in compliant vessels and the numerical technology employed to solve these 1D equations are reported in Müller and Blanco (2015) and Müller et al. (2016a,b).

The ADAN model was simulated for 10 s, and the results of the last cardiac cycle are considered in the comparisons performed in next section.

### 3.3. Open-loop bond graph model vs. ADAN model

**Figure 14:**
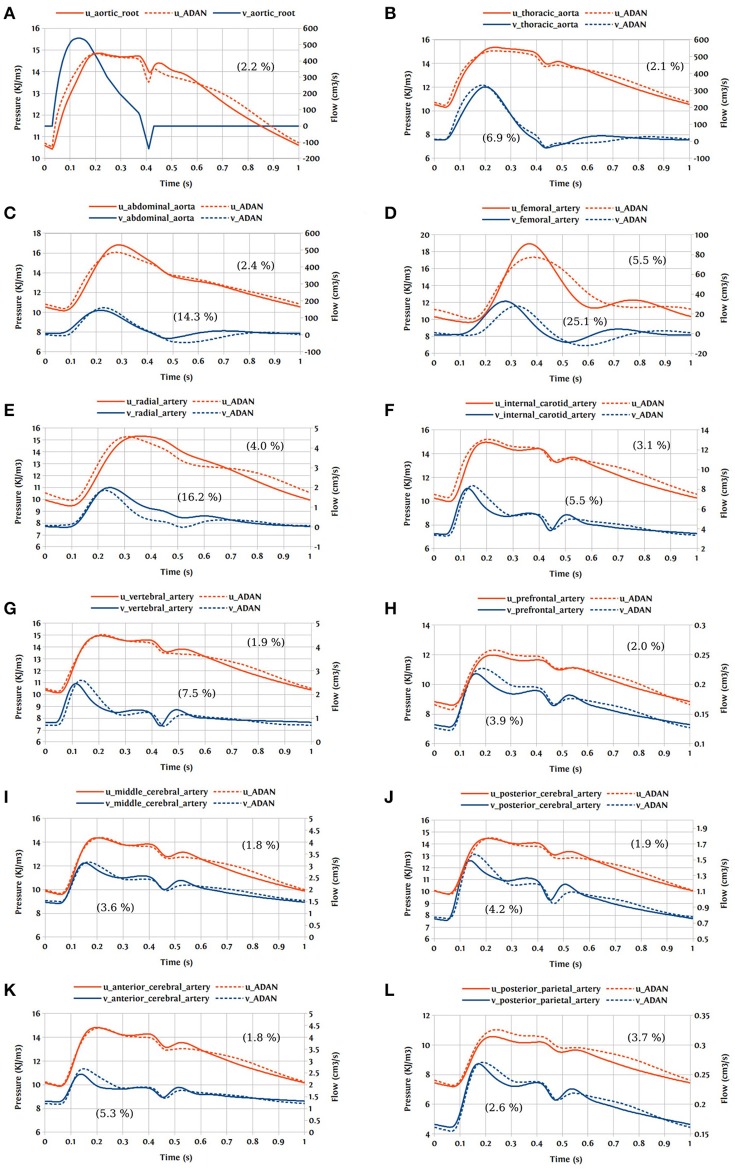
Comparison of pressure and flow waveforms between the bond graph model and ADAN solution; u, pressure; v, flow; **(A)** aortic root; **(B)** thoracic aorta; **(C)** abdominal aorta; **(D)** femoral artery; **(E)** radial artery; **(F)** internal carotid artery; **(G)** vertebral artery; **(H)** prefrontal artery; **(I)** middle cerebral artery; **(J)** posterior cerebral post-communicating artery; **(K)** anterior cerebral artery; **(L)** posterior parietal artery. In parentheses are root mean square errors (RMSE) computed between the simulations and ADAN solution and expressed in percentage relative to the ADAN solution systolic values.

In this first case, we are comparing the results of the bond graph arterial model (open-loop) for ADAN (see Figure 12) with the reference solution provided by the ADAN 1D model. All the parameters incorporated in the bond graph arterial model for this simulation are adopted from the ADAN 1D model. The inflow BC at the aortic root is prescribed using the flow curve shown in Figure 14A acquired from Blanco et al. (2014). Overall, pressure and flow rate waveforms as predicted by the bond graph model aligns closely with the ADAN solution. Qualitative comparisons of pressure and flow waveforms at different arterial locations are given in Figure 14. A quantitative assessment of the relative error is performed by computing the root mean square of the error (RMSE) of the predicted waveforms compared with ADAN solution.

In the main aortic segments, the pressure and flow waveforms are very similar to the ADAN solution in the aortic root, thoracic aorta, and proximal abdominal aorta. In the lower and upper limbs, waveform predictions at the femoral and radial arteries are in agreement with ADAN solution, however by refining the model and increasing the number of bond graph modules in the peripheral arteries we would be able to obtain a better match. Regarding the cerebral circulation, the pressure and flow waveforms predicted by the bond graph model are compared with ADAN solution in the internal carotid artery, vertebral artery, prefrontal artery, middle cerebral artery, anterior cerebral artery, posterior cerebral post-communicating artery, and posterior parietal artery. We observe that, the amplitude and shape of the pressure and flow waveforms in the cerebral arteries are well captured by the bond graph model.

### 3.4. Closed-loop bond graph model

Now the case in which we have a closed-loop bond graph model is simulated (see Figure 10). The model was run for ten cardiac cycles (*T* = 1 s) using a 0.001 s time step, with tolerance 10^−7^ and CVODE solver. For the full model with 258 modules (244 modules for ADAN, 8 modules for the heart, 3 modules for pulmonary and systemic circulation loops), the computation took about 23 s, which is near real-time simulation. With a simpler model (only ADAN-86 without ADAN-brain), the same simulation takes 5 s which is faster than real-time simulation. The simulation time has been measured within OpenCOR, running on a Linux Ubuntu 17.10 machine with Intel^®^ Core i7-6820HK Processor @ 2.70 GHz.

**Figure 15:**
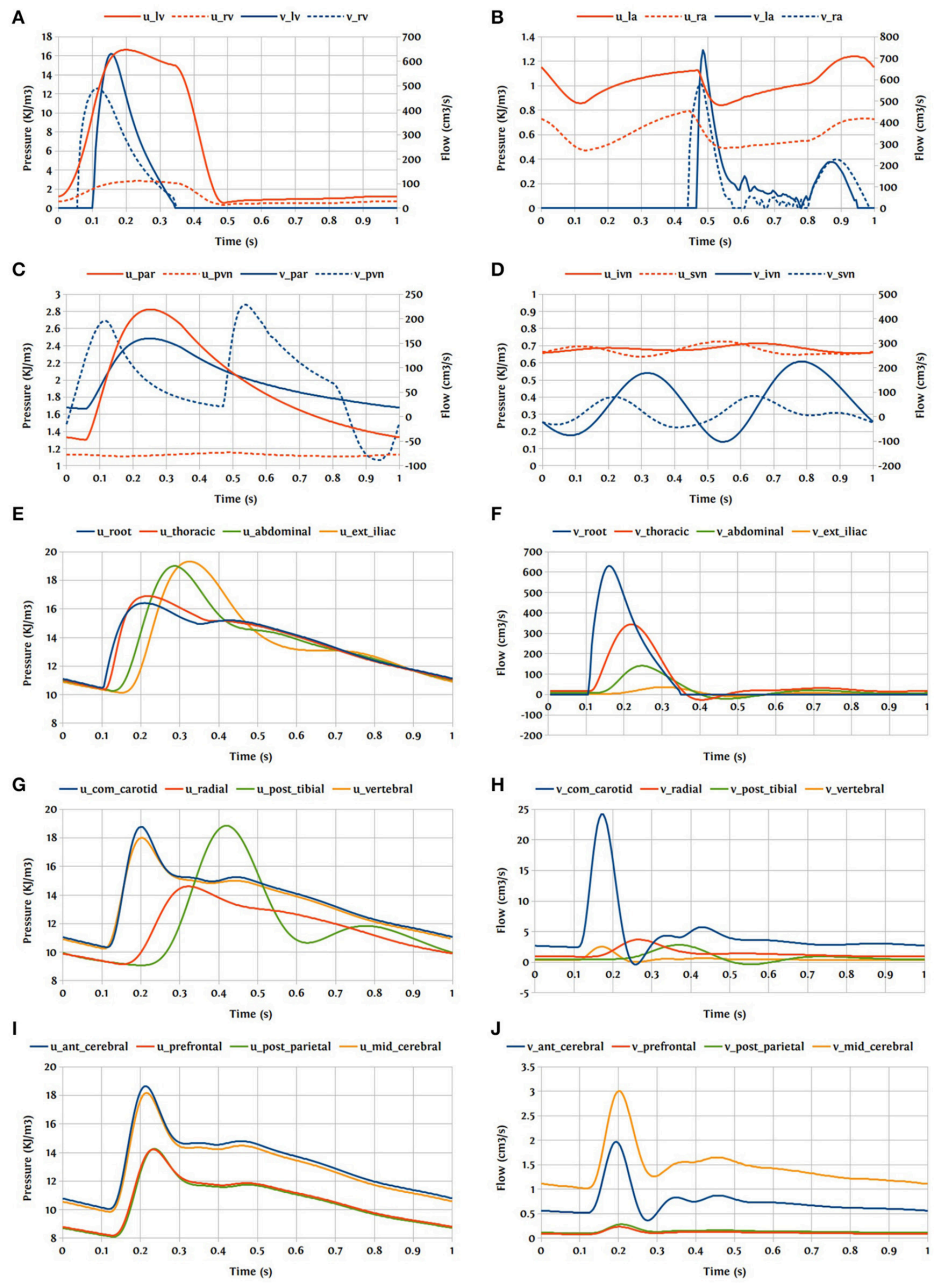
Pressure and flow rate in the main segments during one cardiac cycle; u, pressure; v, flow; **(A)** lv, left ventricle; rv, right ventricle; **(B)** la, left atrium; ra, right atrium; **(C)** par, pulmonary arteries; pvn, pulmonary veins; **(D)** ivn, inferior vena-cava; svn, superior vena-cava; **(E,F)** ext, external; **(G,H)** com, common; post, posterior; **(I,J)** ant, anterior; mid, middle.

One cardiac cycle of the cardiovascular system model already in the periodic state is visualized in Figure 15.

## 4. Discussion and future work

In this paper, we have used the bond graph concept for constructing 0D models. We utilized the bond graph formalism to assemble a system of ODEs in a structured way that satisfies mass and energy conservation for flow in an anatomically detailed model of the cerebral circulation. The most important feature that the bond graph approach provides is the sub-division and reticulation of a network, which is equivalent to the system decomposition or disaggregation, facilitating the introduction/application of hierarchical modeling concepts. We have compared the predicted flow and pressure at a number of points in the vascular model against the solution delivered by a 1D blood flow model and, as an illustrative proof-of-concept, we have shown that the pressure and flow rate waveforms predicted by the bond graph model are within 5% of the 1D model solution at the points of comparison in the cerebral circulation model.

OpenCOR used the CVODE solver which is a solver for stiff and nonstiff ODE systems (initial value problem) given in explicit form *y*′ = *f*(*t, y*). The Backward Differentiation Formula (BDF) is employed as the integration method with a dense direct linear solver for Newton iterations. The bond graph model runs approximately 200x faster than the 1D model and at close to real time on a desktop computer for the level of detail we included. The low computational effort is due to the lumped nature of the mathematical representation provided by the ODE system. There are several simplifying assumptions to derive the lumped parameter models from the Navier-Stokes equations. We considered the fluid as Newtonian and applied a flow profile derived for laminar and stationary flow conditions. We also assumed a constant Young's modulus and a uniform circular cross-section along the length of the segment. The assumption of uniform elastic properties over a large pressure range has some disadvantages. While it is desirable to keep the model linear for speed-up, the drawback is that it is unable to represent the complex behavior of the vessel wall accurately, and therefore affects the model's predictive ability for various pressure levels. This problem can be addressed by incorporating a nonlinear elastic material in the *C*-element that provides the pressure-dependent compliance. The current model has only 86 compartments for systemic flow paths outside the head in addition to the heart and lungs (since we were focussing on the cerebral circulation), but we plan to extend the model to include higher resolution models of the rest of the systemic circulation in the future. We will also look into the possibilities of profiling and parallelising the code for optimisation and speed-up.

Our overall goal for this work has been to create an anatomically detailed model of the cardiovascular circulation that can be made patient-specific (at least to some extent) and can be run in real time (Safaei et al., 2016). The model presented here will be made available in the public domain with freely available open source tools, enabling users to examine pressures and flow rates at any point in the circulation under a variety of physiological conditions. The bond graph formulation makes it straightforward to extend the model to include various tissue exchange mechanisms and to incorporate tissue and cellular parameters that characterize various chronic diseases. In the present contribution, this formalism served to provide a structured and compartmental description of the whole circulatory system including the heart, pulmonary loop and the venous system. This model can also include self-regulating and metabolic dynamics in a simple way and at a low computational cost. Cerebral auto-regulation is a precise system involving vasodilation and vasoconstriction in a network of collateral vessels. By adding metabolic models to the bond graph model we would be able to simulate cerebral auto-regulation, which is a feedback mechanism driving an appropriate blood supply into the cerebral vasculature depending on the oxygen demand by the brain. There are also many other physiological mechanisms that can be added into this system such as baroreflex regulation, respiratory control system, autonomic nervous system, etc.

## Author contributions

SS, PH, and PB contributed conception and design of the study; SS performed the statistical analysis; SS wrote the first draft of the manuscript; SS, PH, PB, LM, and LH wrote sections of the manuscript. All authors contributed to manuscript revision, read and approved the submitted version.

### Conflict of interest statement

The authors declare that the research was conducted in the absence of any commercial or financial relationships that could be construed as a potential conflict of interest.
