# Supplementary material for: Bond Graph Model of Cerebral Circulation: Toward Clinically Feasible Systemic Blood Flow Simulations
Source: Front Physiol. 2018 Mar 2;9:148. doi: 10.3389/fphys.2018.00148 (PMC5841312; doi:10.3389/fphys.2018.00148)
Supplement: Supplementary file 1 [file Tables.pdf]

Supplementary Material: Bond Graph Model of Cerebral Circulation: Toward Clinically Feasible Systemic Blood Flow Simulations

1 SUPPLEMENTARY TABLES

Quantity	Blood Volume	m^3
Potential	Blood Pressure *(Pascal)*	$\mathrm{J.m}^{-3}$
Flow	Blood Flow	$\mathrm{m}^3.\mathrm{s}^{-1}$
Capacitance	Vessel Wall Compliance	$\mathrm{m}^6.\mathrm{J}^{-1}$
Resistance	Blood Viscous Resistance	$\mathrm{J.s.m}^{-6}$
Inertance	Blood Inertia	$\mathrm{J.s}^2.\mathrm{m}^{-6}$
Power	Heart Power	$\mathrm{J.s}^{-1}$

Table S1. Bond graph units in the fluid mechanics domain.

**Bond Graph Element**	**Application**
$\mu\upsilon$-type	single vessel segment
$\upsilon\mu$-type	single vessel segment
$\mu\mu$-type	single vessel segment
$\upsilon\upsilon$-type	single vessel segment
$\mu\upsilon$-split-type	junction (splitting flow)
$\upsilon\upsilon$-merge-type	junction (merging flow)
$\mu\mu$-BC-type	terminal vessel segment

Table S2. The library of bond graph elements in the fluid mechanics domain.

**Part**	**Parameter**	**Value**	**Unit**
Heart	T	1.0	s
	t_{ac}	0.8	s
	t_{ar}	0.97	s
	T_{ac}	0.17	s
	T_{ar}	0.17	s
	T_{vc}	0.34	s
	T_{vr}	0.15	s
	E_{ra}^{A}	$7.998e+6$	$\mathrm{J.m^{-6}}$
	E_{ra}^{B}	$9.331e+6$	$\mathrm{J.m^{-6}}$
	E_{rv}^{A}	$73.315e+6$	$\mathrm{J.m^{-6}}$
	E_{rv}^{B}	$6.665e+6$	$\mathrm{J.m^{-6}}$
	E_{la}^{A}	$9.331e+6$	$\mathrm{J.m^{-6}}$
	E_{la}^{B}	$11.997e+6$	$\mathrm{J.m^{-6}}$
	E_{lv}^{A}	$366.575e+6$	$\mathrm{J.m^{-6}}$
	E_{lv}^{B}	$10.664e+6$	$\mathrm{J.m^{-6}}$
	q_{ra}^{0}	$4.0e-6$	$\mathrm{m^3}$
	q_{rv}^{0}	$10.0e-6$	$\mathrm{m^3}$
	q_{la}^{0}	$4.0e-6$	$\mathrm{m^3}$
	q_{lv}^{0}	$5.0e-6$	$\mathrm{m^3}$
	R_{tr}	$34.6427e-6$	$\mathrm{J^{-0.5}.s^{-1}.m^{4.5}}$
	R_{pu}	$30.3124e-6$	$\mathrm{J^{-0.5}.s^{-1}.m^{4.5}}$
	R_{mi}	$34.6427e-6$	$\mathrm{J^{-0.5}.s^{-1}.m^{4.5}}$
	R_{ao}	$30.3124e-6$	$\mathrm{J^{-0.5}.s^{-1}.m^{4.5}}$
Pulmonary	C_{par}	$0.0309077e-6$	$\mathrm{J^{-1}.m^6}$
	C_{pvn}	$0.60015e-6$	$\mathrm{J^{-1}.m^6}$
	I_{par}	0.0	$\mathrm{J.s^2.m^{-6}}$
	I_{pvn}	0.0	$\mathrm{J.s^2.m^{-6}}$
	R_{par}	$10.664e+6$	$\mathrm{J.s.m^{-6}}$
	R_{pvn}	$1.333e+6$	$\mathrm{J.s.m^{-6}}$
Systemic	C_{svn}	$0.1125281e-6$	$\mathrm{J^{-1}.m^6}$
	R_{svn}	$3.999e+6$	$\mathrm{J.s.m^{-6}}$
	I_{svn}	$0.06665e+6$	$\mathrm{J.s^2.m^{-6}}$
Wall Thickness	a	0.2802	
	b	-505.3	$\mathrm{m^{-1}}$
	c	0.1324	
	d	-11.14	$\mathrm{m^{-1}}$
Voigt Model	f	0.01	s

Table S3. Parameters for the heart, systemic and pulmonary circulation loops.
